# Genome-Wide Analysis of Germline Signaling Genes Regulating Longevity and Innate Immunity in the Nematode *Pristionchus pacificus*

**DOI:** 10.1371/journal.ppat.1002864

**Published:** 2012-08-09

**Authors:** Robbie Rae, Amit Sinha, Ralf J. Sommer

**Affiliations:** Department of Evolutionary Biology, Max Planck Institute for Developmental Biology, Tübingen, Germany; University of Pennsylvania, United States of America

## Abstract

Removal of the reproductive system of many animals including fish, flies, nematodes, mice and humans can increase lifespan through mechanisms largely unknown. The abrogation of the germline in *Caenorhabditis elegans* increases longevity by 60% due to a signal emitted from the somatic gonad. Apart from increased longevity, germline-less *C. elegans* is also resistant to other environmental stressors such as feeding on bacterial pathogens. However, the evolutionary conservation of this pathogen resistance, its genetic basis and an understanding of genes involved in producing this extraordinary survival phenotype are currently unknown. To study these evolutionary aspects we used the necromenic nematode *Pristionchus pacificus*, which is a genetic model system used in comparison to *C. elegans*. By ablation of germline precursor cells and subsequent feeding on the pathogen *Serratia marcescens* we discovered that *P. pacificus* shows remarkable resistance to bacterial pathogens and that this response is evolutionarily conserved across the Genus *Pristionchus.* To gain a mechanistic understanding of the increased resistance to bacterial pathogens and longevity in germline-ablated *P. pacificus* we used whole genome microarrays to profile the transcriptional response comparing germline ablated versus un-ablated animals when fed *S. marcescens*. We show that lipid metabolism, maintenance of the proteasome, insulin signaling and nuclear pore complexes are essential for germline deficient phenotypes with more than 3,300 genes being differentially expressed. In contrast, gene expression of germline-less *P. pacificus* on *E. coli* (longevity) and *S. marcescens* (immunity) is very similar with only 244 genes differentially expressed indicating that longevity is due to abundant gene expression also involved in immunity. By testing existing mutants of *Ppa-*DAF-16/FOXO and the nuclear hormone receptor *Ppa-*DAF-12 we show a conserved function of both genes in resistance to bacterial pathogens and longevity. This is the first study to show that the influence of the reproductive system on extending lifespan and innate immunity is conserved in evolution.

## Author Summary

Removal of the germline in the nematode *Caenorhabditis elegans* can increase lifespan and resistance to bacterial pathogens. Currently there is no information on what genes are regulated to produce this resistance phenotype in other nematodes and whether they are the same as genes involved in lifespan regulation. We used the necromenic nematode, *Pristionchus pacificus*, a species that diverged from *C. elegans* 250–400 MYA, ablated its germline and found increased resistance to the pathogens *Serratia marcescens* and *Xenorhabdus nematophila*. In a novel manner we performed cell ablation of the germline, exposure to bacterial pathogens and used whole genome microarrays of the same animals to find that this resistance is due to expression of genes involved in insulin signaling, nuclear pore complexes, ribosomal translation and lipid production. Furthermore, we see little difference between germline ablated lifespan and immunity leading us to believe that living longer is due to an abundance of genes also being involved with immunity. We could also show that, similar to *C. elegans*, the transcription factor DAF-16/FOXO and nuclear hormone receptor DAF-12, are integral for this response. Our study is the first to understand how the reproductive system regulates both lifespan and innate immunity transcriptionally and offers insights into the signaling cascades involved with resisting pathogen attack.

## Introduction

Removal or alteration of sexual organs can cause a dramatic increase in lifespan in animals including fish, flies, nematodes, mice and humans, but underlying mechanisms remain unknown. For example, gonadectomy slows body wasting and intestinal atrophy in the lamprey *Lampetra fluviatilis* and increases the lifespan in Pacific salmon [1]–[3]. Castration halts major organ degeneration in male marsupial mice [4], [5]. Also, transplantation of ovaries from young mice into older mice and genetic delay of the menopause can increase lifespan and dramatically reduce age related complications, respectively [6], [7]. Even in humans castrated men have a 24% increase in median lifespan compared to un-castrated [8]. In the model organisms *Caenorhabditis elegans* and *Drosophila melanogaster* removal of the germline results in an increase in longevity of 40–60% [9], [10]. This response depends on several genes including DAF-16/FOXO-like transcription factor, the ankyrin repeat KRI-1, the nuclear hormone receptor DAF-12, the cytochrome P450 DAF-9, the transcription elongation factor TCER-1 [11] and processes such as autophagy, and fat metabolism [12]–[14]. However, there has never been a systematic analysis of the whole genome transcriptional response to understand what genes are being expressed to decelerate aging and increase lifespan.

It has been shown in *C. elegans* that genes affecting lifespan also affect other phenotypes such as resistance against bacterial pathogens [15]. Specifically, long-lived *C. elegans* germline deficient animals can survive when fed various pathogens [16]–[19]. However, it is currently unknown how conserved this response of germline ablation-induced longevity and pathogen resistance is in other free-living nematodes and more distantly related animals. The diplogastrid nematode *Pristionchus pacificus* diverged from *C. elegans* 250–400 million years ago [20] and is used as a comparative model to *C. elegans*. This comparative research has revealed evolutionary changes in vulva development [21], gonad morphogenesis [22], and chemosensory behavior [23] compared to *C. elegans*. The toolkit for *P. pacificus* research includes a fully sequenced genome and a well characterized proteome [20], [24], forward and reverse genetics, transgenic techniques [25], full genome microarray technology [26] and hundreds of naturally isolated *P. pacificus* strains isolated from around the world [27]. Interestingly, *C. elegans* and *P. pacificus* also differ in their ecological niches. *C. elegans* can be isolated from compost heaps, snails and rotten fruits [28], whereas *P. pacificus* is usually isolated from a range of scarab beetles [29]. *P. pacificus*, as well as other *Pristionchus* species lives in a necromenic lifestyle, that attach to passing beetles as dauers and feed on the plethora of microorganisms growing on the cadaver when the beetle dies [30].

The comparison of pathogen resistance of germline-ablated *P. pacificus* and *C. elegans* is of special interest given the different response of these two nematodes to bacteria under normal growth conditions. Specifically, *P. pacificus* can feed on *Pseudomonas aeruginosa*, *Staphylococcus aureus*, *Bacillus thuringiensis* Cry 5B toxin and *B. thuringiensis* DB27, whereas *C. elegans* dies on these bacterial strains [30]–[32]. There are six signaling pathways that have been identified as being critical for *C. elegans* survival when fed an array of bacterial and fungal pathogens e.g. ERK MAP kinase, p38 MAP kinase, TGF β, programmed cell death, DAF-2/DAF-16 insulin-like receptor signaling and JNK-like MAP kinase [33]. As some of these pathways also regulate aging in *C. elegans* (e.g. FOXO/DAF-16 insulin-like signaling [15]) it remains to be seen how these animals either use shared or distinct mechanisms to regulate innate immunity and aging.

Here, we investigated whether germline manipulation in *P. pacificus* would increase survival when fed the opportunistic human pathogen *Serratia marcescens*, whether this response is evolutionarily conserved across the Genus *Pristionchus*, and carried out a detailed analysis of the transcriptional mechanistic processes governing this response. Using whole genome microarrays comparing unablated and germline ablated *P. pacificus* we found that resistance to pathogenic bacteria is due to differential expression of genes involved in insulin signaling, pathogen response, lipid metabolism, and core cellular processes like ribosomal translation, proteasome function, nuclear pore complexes. Furthermore, we show that germline ablations of *P. pacificus daf-16* and *daf-12* mutants severely affect the survival when fed bacterial pathogens, thereby underpinning the importance of insulin signaling. Our study is the first to provide an understanding of how the reproductive system regulates both lifespan and innate immunity transcriptionally and offers insights into the signaling cascades involved with resisting pathogen attack.

## Results/Discussion

### Germline ablated *P. pacificus* are resistant to bacterial pathogens

**Figure 1:**
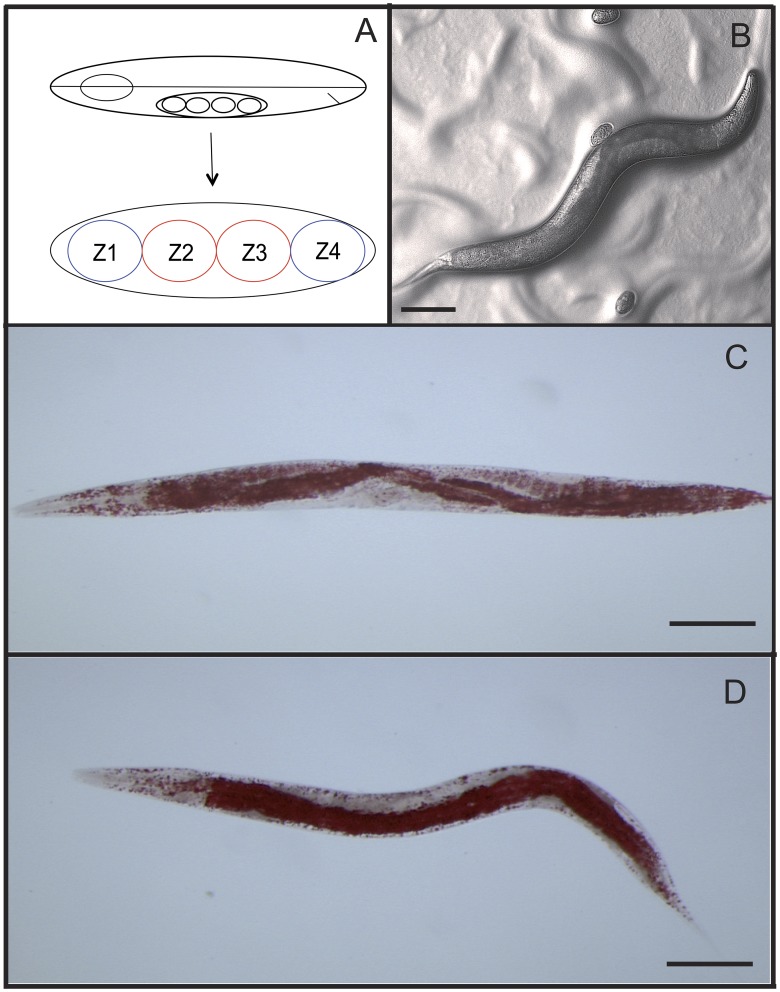
Effect of cell ablation on *P. pacificus*. Z2 and Z3 give rise germline and Z1 and Z4 give rise to somatic gonad (A). *P. pacificus* WT un-ablated (B). *P. pacificus* WT stained with Oil red O showing tryglyceride staining of intestine, hypodermis, gonad and eggs (C) and *P. pacificus* Z2 and Z3 and ablated similarly stained showing concentration of triglycerides in intestine, like germline ablated *C. elegans* (46) (D). Scale bar represents approx. 100 µm.

As in *C. elegans* the gonad of *P. pacificus* consists of four cells (Z1, Z2, Z3 and Z4) with Z2 and Z3 giving rise to the germline and Z1 and Z4 making the somatic gonad, which can be removed using laser microsurgery (Figure 1 a) [34]. Previously it was shown that *P. pacificus* (Figure 1b) is long lived when the germline is ablated [9], [35]. We were interested to see if survival on bacterial pathogens would also be enhanced. We ablated the germline (Figure 1c,d) and the somatic gonad separately and fed *S. marcescens. S. marcescens* can be isolated from soil, insects and *Pristionchus* nematodes emerging from beetles and is lethal to both *P. pacificus* and *C. elegans* [30].

**Figure 2:**
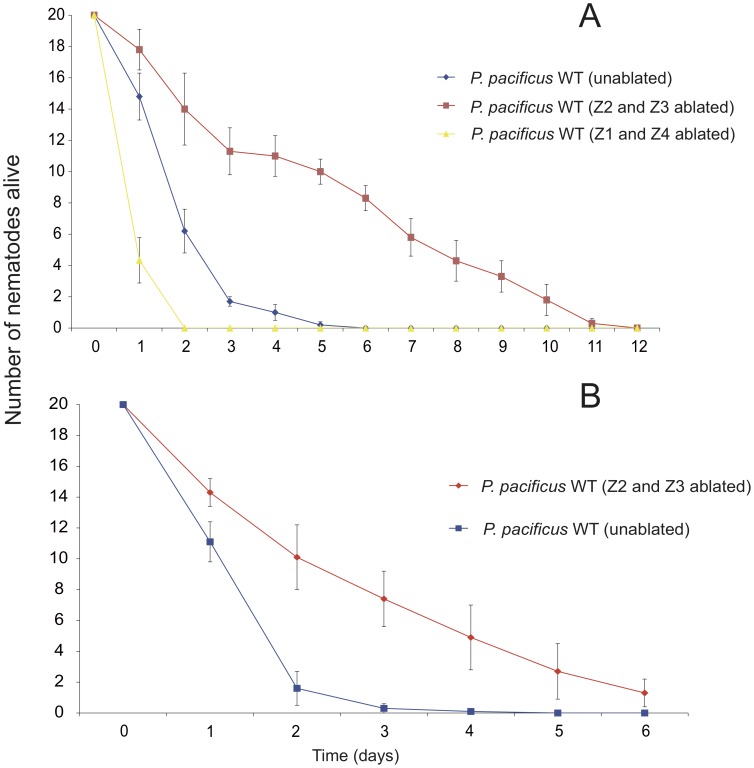
Effect of ablation of survival of *P. pacificus* fed bacterial pathogens. Number of alive *P. pacificus* WT (blue), Z2 and Z3 ablated (red) and Z1 and Z4 (yellow) exposed to *S. marcescens* (A) and *X. nematophila* (B). Batches consisting of twenty nematodes were added separately to three plates and survival was monitored daily beginning on day 0. Error bars represent ± S.E.M.

Germline ablated *P. pacificus* survive significantly longer than un-ablated nematodes on *S. marcescens* (Figure 2a) (P<0.001, Supplementary Table S1). In contrast, ablation of Z1 and Z4 does not cause resistance to *S. marcescens* indicating that neither ablation nor sterility *per se* contributes to increased resistance (Figure 2a). Also, the effect is not limited to one pathogen as germline ablated *P. pacificus* also survive on the entomopathogenic nematode associated bacterium *Xenorhabdus nematophilum* that kills wild type *P. pacificus* in two days (Figure 2b). Thus, similarly to germline loss induced longevity, *P. pacificus* germline ablated nematodes are resistant to diverse bacterial pathogens.

It must be noted that in *C. elegans*, when Z1 and Z4 are ablated, the germline precursor cells (Z2 and Z3) will die [34]. Death of Z2 and Z3 is also observed upon Z1 and Z4 ablation in *P. pacificus*, except in a small fraction of animals that develop germline tumors at a rate of about 10% (e.g. 4 out of 35 animals, as reported in [22]).

### Germline ablated resistance to *S. marcescens* is conserved across the Genus *Pristionchus*

**Figure 3:**
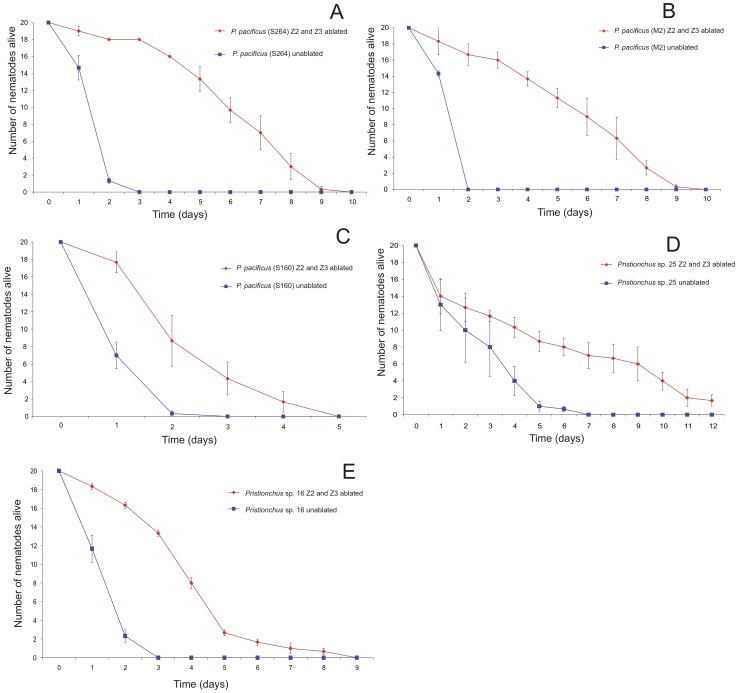
Effect of ablation of survival of *Pristionchus* species and strains exposed to *S. marcescens*. Survival of (A) *P. pacificus* S264 Z2 and Z3 ablated (red) and un-ablated (blue), (B) *P. pacificus* M2 Z2 and Z3 ablated (red) and un-ablated (blue), (C) *P. pacificus* RS5160 Z2 and Z3 ablated (red) and un-ablated (blue), (D) *Pristionchus* sp. 3 Z2 and Z3 ablated (red) and un-ablated (blue), (E) *Pristionchus* sp. 16 Z2 and Z3 ablated (red) and un-ablated (blue). Error bars represent ± S.E.M.

Currently, over 400 strains of *P. pacificus* isolated worldwide [27] are available in a collection in the Sommer lab in Tuebingen, Germany. Therefore, to investigate if this bacterial resistance response was only present in the *P. pacificus* wild type strain PS312, we chose strains from Montenegro (M2), China (S264) and Japan (RS5160), ablated their germline precursor cells and fed them *S. marcescens* following germline ablation All three *P. pacificus* strains also show significant resistance to *S. marcescens* demonstrating the evolutionary conservation of somatic gonad signaling contributing to innate immunity across *P. pacificus* species (Figure 3 a–c). To investigate this further we expanded to two other *Pristionchus* species from a group of 25 *Pristionchus* species isolated from around the world, available in the Tuebingen collection. We chose *Pristionchus* sp. 3 and *Pristionchus* sp. 16 and repeated our previously described experiment. Again, we observe increased resistance to *S. marcescens* dependent on a signal from the somatic gonad when the germline is ablated. Both species survive significantly longer than un-ablated animals (Figure 3 d, e).

### Transcriptional responses of germline ablated and un-ablated *P. pacificus* exposed to *E. coli* and *S. marcescens*

Results from germline ablation experiments indicate that the germline regulates some longevity and immunity related signals in a cross talk with the somatic cells of the animal. In order to gain a mechanistic understanding of what genes are regulated when the germline precursor cells (Z2 and Z3) are removed and nematodes are fed pathogens, we assessed the transcriptional response using whole genome microarrays. Many studies in *C. elegans* have looked for genes that mediate the enhanced longevity phenotypes of germline-less animals via RNAi screening [13], [14], [36]. Here, we have taken an unbiased approach to identify the set of all genes regulated in response to germline ablation, and tried to identify which of them might be functionally relevant. To our knowledge this is the first attempt to couple experimental cell ablation followed by pathogen exposure to microarray analysis.

**Figure 4:**
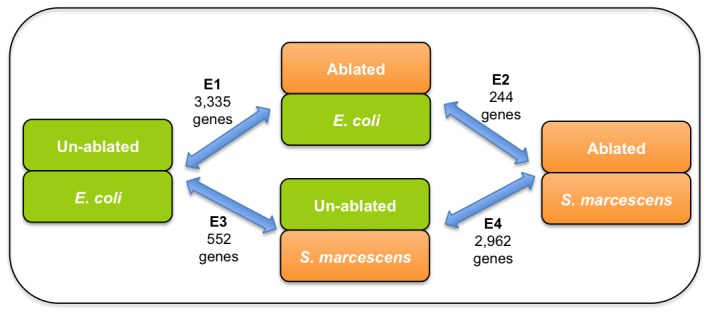
Enhanced longevity due to germline ablation is the major transcriptional component of increased pathogen resistance of these animals. In this schematic of microarrays comparisons, each experiment is represented by two parameters - the ablation status and the bacteria fed, while the double arrows show the samples co-hybridized on the same array. In the first experiment E1, 3,335 genes were differentially expressed when germline ablated animals fed on *E. coli* were compared against un-ablated animals also fed on *E. coli*. In second experiment, E2, only 244 genes were differentially expressed when ablated animals exposed to *S. marcescens* were compared against ablated animals exposed only to *E. coli*, thus indicating that effects of germline ablation are the major transcriptional component towards increased lifespan and pathogen resistance. We also compared these data to transcriptional response of wild-type *P. pacificus* worms exposed to *S. marcescens* (experiment E3, 552 genes, AS, RR, II and RJS, unpublished data). Using these data, we calculated the comparison E4 between ablated versus unablated animals, both fed on *S. marcescens*, and found 2,962 differentially expressed genes.

In the first set of experiments, to identify longevity regulating genes, we ablated the *P. pacificus* germline precursor cells and compared them to un-ablated animals, feeding both of them on *E. coli* OP50. In the second set of experiments, we looked at the pathogen response of germline-ablated animals fed on *S. marcescens* in comparison to germline-ablated animals fed on the lab food source *E. coli* OP50, to check whether long-lived animals require additional transcriptional activity to defend against a pathogen (see experimental design in Figure 4). For each condition, we used four independent biological replicates of a pool of about 100 animals each that were either ablated or unablated and exposed either to the pathogen *S. marcescens* or the control food source *E. coli* OP50 for four hours in our microarray experiments. We chose the standard lab food bacterium *E. coli* OP50 as the baseline food source to monitor lifespan so as to enable direct comparisons with various *C. elegans* studies that have also used *E. coli* OP50 as the standard food source [12]–[16]. Similarly, for survival assays on the pathogen *S. marcescens*, we again used *E. coli* OP50 as the control food source, as is typical in such studies in *C. elegans* [15], [16]. In all experiments, young-adult *P. pacificus* animals were exposed to the respective bacterium for 4 hours, a time point when immediate-early pathogen response genes can be robustly detected and the expression profile is relatively unaffected by secondary effects of pathogenesis (AS, RR, II and RJS, unpublished data).

In the comparison of germline-ablated adults versus the un-ablated controls fed on *E. coli* OP50, we find 3,335 genes to be differentially expressed at a FDR corrected p-value threshold of 0.05 and a log2 gold change cut-off at 1.5 (corresponding to a relative fold change of about 2.8 on an absolute count scale, Expression profile E1 in Figure 4, Supplementary Table S2). Interestingly, in the second experiment, comparing germline ablated *P. pacificus* fed *E. coli* OP50 or *S. marcescens*, we found only 244 genes to be differentially expressed (Expression profile E2 in Figure 4, Supplementary Table S3). The relatively small number of genes regulated by pathogen exposure indicates that the contribution of germline-ablation to enhanced longevity is the major component explaining their increased pathogen resistance. Thus, we characterized the expression profile from the first experiment in more detail, as described below.

#### a) Differential regulation of translation elongation and initiation and protein homeostasis in long-lived animals

Out of the ∼3000 genes differentially expressed upon germline ablation, some would have a role in longevity extension, while others would be related only to the development of the germline. In agreement with this expectation, the most enriched biological processes in a Gene Ontology (GO) analysis (Supplementary Table S4a) are related to “determination of adult lifespan” or to germline and reproductive development (e.g. “hermaphrodite genitalia development”, “germ cell development”, “gonad development”). The differentially expressed gene set is also highly enriched for genes that encode for “structural constituent of ribosome” (Supplementary Table S4b) and that localize predominantly to the “ribosome”, “small ribosomal subunit” and “ribonucleoprotein complex” (Supplementary Table S4c), while the molecular functions “translation initiation factor activity” and “translation elongation factor activity” are also significantly enriched (Supplementary Table S4b). These observations suggest that regulation of translation initiation and elongation at the ribosome is an important component of longevity enhancement program in *P. pacificus*. This interpretation also agrees with the findings in *C. elegans*, whereby the transcription elongation factor TCER-1 was found to be integral for longevity induced by ablation of the germline and to be essential for completion of RNA synthesis during gene expression [36]. Thus, the molecular components regulating germline appear to be conserved between *C. elegans* and *P. pacificus*.

#### b) Proteasome core subunits and cytoplasmic chaperonins are misregulated in germline-ablated animals

Regulation of protein homeostasis is also a key aspect of lifespan maintenance in *C. elegans* [37]. In our GO analysis of *P. pacificus* data, we find significant enrichment of terms related to proteasome function, such as “proteasome core complex”, “protein folding”, “protein refolding” (Supplementary Table S4a) and “unfolded protein binding” (Supplementary Table S4b). Further, the most enriched Pfam domains in the genes regulated by germline include the category “Proteasome” and other domains related to proteasome function (“ubiquitin”, “PCI” and “Mov34”, Supplementary Table S5). The eukaryotic 26S proteasome comprises a core 20S subunit and regulatory 19S regulatory subunits. We observe that upon germline-ablation, five of the seven subunits of the proteasome core alpha subunit (*pas* gene family), and six of the seven subunits of the beta subunit (pbs gene family) are significantly downregulated transcriptionally. Loss of function of any of the components of the proteasome core subunit is known to activate SKN-1 dependent oxidative stress and detoxifying response in a feedback loop [38]. The activation of SKN-1 in turn is responsible for enhanced longevity of insulin signaling mutants in parallel to the *Cel-*DAF-16 pathway [39]. Disruption of proteasomal function has also been shown to increase pathogen resistance [40], [41]. Hence it is likely that the observed downregulation of proteasome core subunit components leads to SKN-1 activation resulting in regulation of expression, which contributes to the enhanced longevity of germline-ablated animals.

We further observed that all eight of the *cct* gene family members in *P. pacificus* are downregulated in germline-ablated animals. The loss of function of cytoplasmic chaperonin complex components in *C. elegans* also leads to SKN-1 activation [38] and loss of function of its components such as *Cel-cct-4* and *Cel-cct-6* has been linked to enhanced longevity phenotypes via SKN-1 activation [42]. Hence the observed downregulation of all *cct* family genes in germline abated *P. pacificus* suggests a causal link to enhanced longevity, presumably via SKN-1 activation. Also, it was recently shown that RNAi inactivation of *C. elegans* genes involved with essential processes such as translation, respiration and protein turnover can result in repulsion of nematodes from normally attractive bacteria [40] and that translational inhibition can activate the immune response [43], [44].

#### c) Potential role for nucleolar and nuclear pore complex components in longevity enhancement

We observe downregulation of *Ppa-nol-5*, *Ppa-nol-6*, and *Ppa-nol-10*, which encode three out of six members of nucleolar RNA associated protein (NRAP) family. Inactivation of a *Cel-nol-6* not only affects ribosome biogenesis but also reduces intestinal pathogen accumulation resulting in enhanced pathogen resistance, by inhibiting the p53 homolog *Cel-cep-1* [45]. However, its role in lifespan regulation has not yet been characterized. Interestingly, RNAi inactivation of *Cel-nol-1*, another member of the same gene family, has independently been shown to enhance lifespan [46]. We thus expect the downregulation of *Ppa-nol-5*, *Ppa-nol-6* and *Ppa-nol-10* to contribute to the enhanced longevity of germline-ablated animals.

Intriguingly, we also find significant downregulation of 14 members of the nuclear pore complex protein family (*npp*) in the germline-ablated animals. There are 21 nuclear pore complex proteins in *C. elegans*, and 15 orthologs have been identified in the latest *P. pacificus* gene annotations so far (www.pristionchus.org). We were surprised to find that all the components of an essential nuclear pore complex are transcriptionally downregulated in long-lived animals. One possibility is that all of them are coordinately regulated by common factors, and the mis-regulation of these components (or their upstream regulator) activates certain stress resistance pathways that ultimately result in enhanced longevity.

#### d) Role of lipid metabolism pathways in enhanced lifespan of germline ablated animals

Given the role of fatty acid desaturation in *C. elegans* longevity [14], we looked into regulation of fatty acid desaturase enzymes in germline ablated *P. pacificus*. While the *C. elegans* genome contains nine genes that encode a protein with a fatty acid desaturase enzyme domain (Pfam name FA_desaturase, Pfam ID = PF00487), we found 17 proteins with this domain in the predicted proteome of *P. pacificus*. One of these genes was robustly upregulated upon germline ablation in *P. pacificus* and shows the highest sequence similarity to *Cel-fat-7*. Fatty acid elongases such as *Cel-elo-2* also regulate lipid composition and lifespan in *C. elegans* [47]. Interestingly, we observe downregulation of the *P. pacificus* ortholog *Ppa-elo-1* in germline ablated animals, suggesting that the role of lipid metabolic pathways in lifespan regulation may also be conserved in *P. pacificus*. Indeed upon ablation of Z2 and Z3 in *P. pacificus* and subsequent staining with Oil Red O, we observe strong localization of triglycerides in the intestine (Figure 1c,d), indicating differences in fat content compared to unablated animals, similar to that seen in germline deficient *C. elegans* [14], [48].

#### e) Enrichment of DAF-16/FOXO targets, dauer regulated genes, and genes regulated in response to various pathogens

Since the DAF-16/FOXO mediated pathway and the TGF-beta pathway play a role in lifespan regulation and innate immunity in *C. elegans* [9], [35], we analyzed the extent and significance of overlap between our differentially expressed genes and the orthologous genes known to be regulated by each of these pathways in *C. elegans*. We indeed observe significant overlap between genes upregulated upon germline ablation and genes regulated by DAF-16 (“Class1” genes from [49], see Table 1), and between genes downregulated upon germline regulation and genes downregulated by TGF-beta ligand DBL-1 (gene set derived from [50], see Table 1), indicating that regulation of these two pathways plays an important role in lifespan regulation in germline-ablated *P. pacificus*.

**Table 1:** Microarray expression clusters showing significant overlap with genes up or down regulated upon germline ablation in *P. pacificus*.

DAF-16, TGF-beta and Dauer related clusters		
Expression cluster	sigScore.UP	sigScore.DOWN
Murphy_etal_cgc5976_Class1	1.35	0
Murphy_etal_cgc5976_Class2	0	1.55
Sinha_etal_ppa_dauers_UP	4.97	0
Sinha_etal_ppa_dauers_DOWN	29.91	302.83
Roberts_etal_2010_DBL-1-DOWN	0	49.64
Roberts_etal_2010_DBL-1-UP	0	12.24

Immunity and Pathogen response related clusters		
Expression cluster	sigScore.UP	sigScore.DOWN
Kao_etal2011_kgb1_regulated	0	27.03
Kao_etal2011_sek1_regulated	0	26.42
Sinha_et_al_serratia_ppa_up*	43.16	0
Sinha_et_al_serratia_ppa_down*	0	275.3
Sinha_et_al_xeno_ppa_up*	57.95	0
Sinha_et_al_xeno_ppa_down*	0	302.83
Sinha_et_al_staph_ppa_up*	37.5	0
Sinha_et_al_staph_ppa_down*	1.45	0
Sinha_et_al_bac27_ppa_up*	9.16	0
Sinha_et_al_bac27_ppa_down*	4.8	0

Germline and reproductive development related		
Expression cluster	sigScore.UP	sigScore.DOWN
cgc6390-oogenesis-enriched	0	190.3
cgc6390-spermatogenesis-enriched	47.63	0
WBPaper00037611:GLD-2-associated	0	41.04
WBPaper00037611:RNP-8-associated	0	59.01

Significance scores are −log10 of the p-values obtained in a 2×2 Fisher's exact test, and have been set to zero in case of non-significant enrichment where p-value >0.05.

* Expression clusters derived from our as yet unpublished data on pathogen response of wild-type *P. pacificus* to *Serratia marcescens*, *Xenorhabdus nematophila*, *Staphylococcus aureus*, and *Bacillus thuringiensis* DB27 (AS, RR, II and RJS, manuscript submitted).

Since, dauers represent a stress-resistant long-lived stage in nematodes like *C. elegans* and *P. pacificus*, we checked for overlap of our data with the transcriptome data of *P. pacificus* dauers [26]. The dauer-regulated genes in *P. pacificus* are significantly over-represented in our set of germline-ablation regulated genes, suggesting that a common module of longevity regulating genes is activated in both dauers and germline-ablated animals.

We have recently characterized pathogen response genes in *P. pacificus* in response to fur different pathogens namely *Bacillus thuringiensis*, *Staphylococcus aureus*, *Serratia marcescens* and *Xenorhabdus nematophila* (AS, RR, II and RJS, unpublished data). Interestingly, we found extensive and highly significant overlap between the genes regulated upon germline ablation and the pathogen response genes known in *P. pacificus* (our unpublished data), particularly those regulated by exposure to gram-negative pathogens *S. marcescens* and *X. nematophila* (Table 1). Further, homologues of genes regulated by the p38 MAPK *sek-1* and the JNK MAPK *kgb-1* in *C. elegans* [51] are also over-represented in the germline ablation dataset (Table 1). These observations suggest that the germline ablation leads to activation of a significantly large number of pathogen response genes, which might be the reason for their enhanced pathogen resistance as well.

Other relevant gene expression clusters that were significantly enriched in the set of differentially expressed genes included those involved in germline development [52], [53] in *C. elegans* (Table 1). This suggests that our expression cluster enrichment analysis is indeed able to capture biologically relevant expression patterns.

#### f) Regulation of antimicrobial response genes

We also find significant upregulation of the antimicrobial lysozyme *Ppa-lys-7*, whose corresponding ortholog, *Cel-lys-7* is a known DAF-16 target [49] and is induced upon *S. marcescens* infection [54]. Similarly, genes encoding several other potential antimicrobial proteins such as the lectins *Ppa-clec-1*, *Ppa-clec-149*, *Ppa-clec-160* and *Ppa-clec-41* are also significantly upregulated upon germline ablation. Thus it appears that ablation of the germline also results in constitutive activation of various components of pathogen response machinery, which potentially contributes to longevity as well as their enhanced pathogen resistance.

#### g) *Ppa-age-1* is downregulated in germline-ablated animals in *P. pacificus*

Apart from this system level analysis of differentially expressed genes, we also looked for regulation of genes that have a known role in longevity in *C. elegans*. Interestingly, we observed significant downregulation of the *P. pacificus* homolog of the *C. elegans* PI3 Kinase subunit *age-1*. *age-1* is a downstream component of insulin signaling whose loss of function leads to increased lifespan in a DAF-16/FOXO dependent manner, as well as increased survival in the presence of pathogenic bacteria [15]. We thus expect the downregulation of *Ppa-age-1* in germline-ablated *P. pacificus* animals to be a major contributor to their extended lifespan and enhanced pathogen resistance.

### Immune response of germline ablated animals to the pathogen *S. marcescens*

Since germline ablated animals also have an enhanced resistance to pathogens in addition to enhanced longevity, we wanted to ascertain whether this enhanced resistance is a separable component from enhanced longevity. We thus, exposed the germline-ablated adults of *P. pacificus* either to the pathogen *S. marcescens* or to the control lab food, *E. coli* OP50, for four hours and compared the transcriptional differences. Although germline ablation results in mis-regulation of a large number of genes (∼3,330, see previous section), the pathogen response of ablated worms comprises only 244 differentially regulated genes (using the same p-value and fold-change cut-offs as in germline ablation effect experiments).

The differential expression of a relatively smaller number of genes in ablated animals exposed to pathogen suggests that the genes differentially expressed due to ablation alone might be sufficient not only for enhanced longevity but also for enhanced pathogen resistance. It is also possible that although the number of regulated genes is small in absolute number, they may still have large phenotypic effects on pathogen resistance, a possibility that awaits functional validation.

Interestingly, of the 244 genes regulated in ablated animals in response to pathogen, only 54 have a corresponding ortholog in *C. elegans*. We find the *P. pacificus* ortholog of the stress-responsive transcription factor *Cel-pqm-1* to be significantly upregulated, implicating activation of the stress response pathway in response to the pathogen. We also observe upregulation of the lectins *Ppa-clec-6*, *Ppa-clec-41*, *Ppa-clec-160*, but surprisingly we do not see induction of any lysozymes. Since *Ppa-lys-7* was already highly induced in germline-ablated animals (see previous section), it is plausible that no further induction of such genes is needed to counter the pathogens. We indeed observe that although there is no differential expression, the absolute expression levels of *Ppa-lys-7* are relatively quite high in these animals (log2(Average_expression) >13.5, Supplementary Table S3). Many genes involved in lipid metabolism such as *Ppa-fat-7*, *Ppa-elo-1, Ppa-idh-1, Ppa-alh-4, Ppa-acs-14* and *Ppa-ech-6* are also downregulated when ablated worms are exposed to *S. marcescens*.

In a previous set of experiments, we have characterized the expression profile of wild-type, un-ablated *P. pacificus* worms in response to the pathogen *S. marcescens* (AS, RR, II and RJS, unpublished data), where we find 552 genes to be differentially expressed, using the same statistical cut-offs of FDR corrected p-value <0.05 and absolute log2(FoldChange) >1.5). Thus the pathogen response of germline-ablated worms (244 genes) is also relatively smaller than that of the wild-type worms. Nonetheless, we find 101 genes to be commonly regulated between the two data sets, the overlap being highly significant (Fisher's exact test p-value = 4.34E-94). The set of overlapping genes include the upregulated genes such as lectin *Ppa-clec-41* and the stress responsive transcription factor *Ppa-pqm-1*, and downregulation of lipid metabolic genes *Ppa-fat-7, Ppa-acs-14, Ppa-alh-4, Ppa-idh-1* and *Ppa-elo-1* (Supplementary Table S6). Thus, the regulation of these genes appears to be crucial for enhanced longevity and pathogen resistance of ablated as well as un-ablated animals. Taken together, our analysis, combining cell ablation, pathogen exposure and microarray analysis in a single experiment, suggests a substantial overlap between lifespan extension and pathogen response in *P. pacificus*.

### Effect of different bacteria on transcriptional differences between ablated and unablated worms

While measuring the transcriptional changes in ablated worms exposed to *S. marcescens* versus ablated worms exposed to *E. coli*, it is possible that some of the differences might not be due to pathogenicity factors but due to inherent differences in the two species of the bacteria used (e.g. nutritional differences between *E. coli* and *S. marcescens*). To characterize such differences, we derived the expression profile of ablated worms versus unablated worms when both are exposed to *S. marcescens* for 4 hours (Experiment E4 in Figure 4, see Methods). We found this expression profile (Supplementary Table S7) to be qualitatively quite similar to the longevity expression profile from the comparison of ablated worms versus unablated worms when both are exposed to *E. coli* for 4 hours (Experiment E1 in Figure 4). Specifically, the fold changes of each gene across the two profiles show an almost perfect correlation (Pearson correlation 0.9, Spearman rank correlation = 0.89, Supplementary Figure S2). Given the excellent correlation between the fold changes across the two profiles E1 and E4, we calculated the overlap between all significantly differentially expressed genes across the two conditions, irrespective of the fold-changes, and found only 292 genes expressed exclusively upon exposure to *S. marcescens* but not on *E. coli* (Supplementary Table S8). Sixty-seven of these genes have a *C. elegans* homolog and belong to diverse functional classes, but do not have any obvious or known role in response to pathogens.

This extensive overlap with the longevity profile E1 includes downregulation of *Ppa-age-1*, components of cytoplasmic chaperonin complex (*cct*- family), genes regulating translation elongation and those involved in proteasomal function. Similarly, this gene set is also enriched for orthologs of DAF-16 targets known from *C. elegans*, as well as other genes involved in the pathogen response in *P. pacificus* (Supplementary Table S9).

### The FOXO transcription factor DAF-16 and nuclear hormone receptor DAF-12 are responsible for increased bacterial resistant in germline ablated *P. pacificus*

In *C. elegans* it has previously been demonstrated that DAF-16 and DAF-12 are responsible for the germline ablated induced longevity in *C. elegans* [9] and in the increased survival of *glp* (germline proliferation) mutants when fed various pathogens [16]–[19], although this depends on pathogen growth conditions [16].

Our microarray data analysis suggests a role for *Ppa-*DAF-16/FOX transcription factor and *Ppa-*DAF-12 nuclear hormone receptor in the enhanced longevity and pathogen resistance observed upon germline ablation. First, we found *Ppa-age-1* to be downregulated in germline ablated animals, which is expected to activate DAF-16 dependent transcriptional activity. Second, we found an enrichment of DAF-16 target genes in germline ablated *P. pacificus* (Table 1). Third, we also observe a significant overlap between germline-regulated genes and genes regulated in *P. pacificus* dauers (Table 1), and *Ppa-daf-16* and *Ppa-daf-12* have already been shown to be key regulators of dauer formation [55], [56]. Hence, all this evidence combined together implicates *Ppa-daf-16* and *Ppa-daf-12* in germline-loss dependent increase in longevity and pathogen resistance.

**Figure 5:**
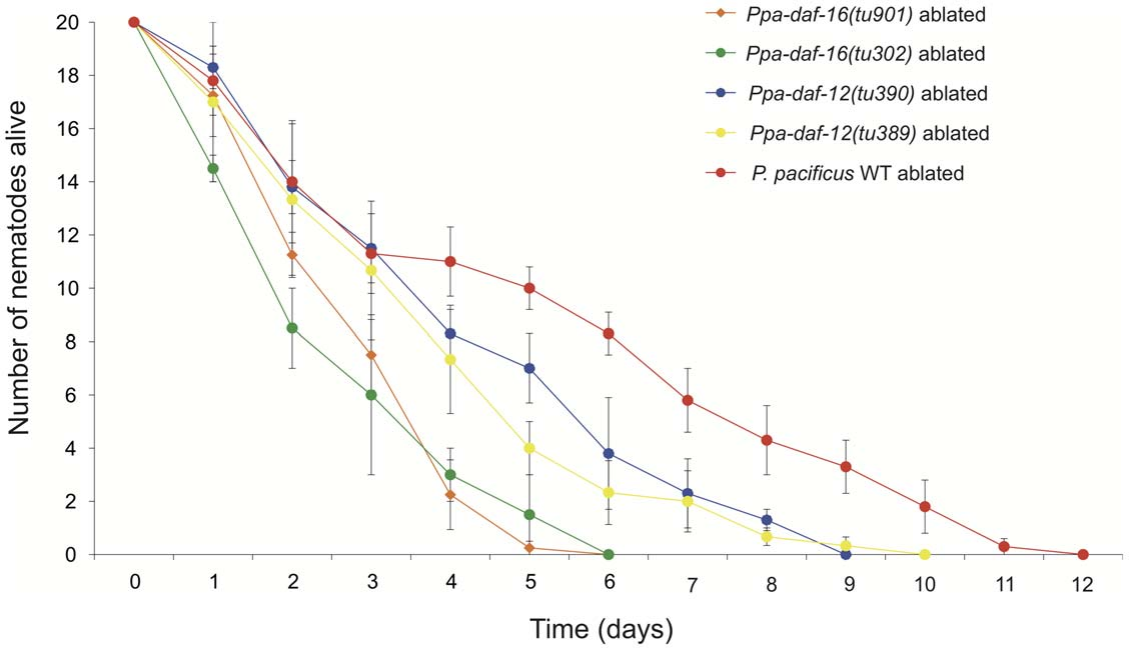
Effect of Z2 and Z3 germline ablation on survival of *P. pacificus* insulin signaling mutants exposed to *S. marcescens*. Survival of Z2 and Z3 ablated *P. pacificus* WT (red), *Ppa-daf-16* (*tu901*) (orange), *Ppa-daf-16* (*tu302*) (green), *Ppa-daf-12* (*tu390*) (blue) and *Ppa-daf-12* (*tu389*) (yellow). Error bars represent ± S.E.M.

As a functional test for the roles of *Ppa-daf-16* and *Ppa-daf-12* in these processes, we ablated the germline precursor cells of two alleles of *Ppa-daf-16* (*tu302* and *tu901*) and *Ppa-daf-12* (*tu390* and *tu389*), as well as a double mutant of both genes and assayed their survival when exposed to the pathogen *S. marcescens*. Both the alleles of germline ablated *Ppa-daf-12* (*tu390* and *tu389*) showed significantly less resistance to *S. marcescens* than germline ablated *P. pacificus* wild type animals (P<0.001) (Figure 5). This was even more apparent when the germlines of *Ppa-daf-16* (*tu302* and *tu901*) were ablated, as they show very weak resistance compared to the wild type *P. pacificus* (P<0.001). Thus, like longevity in *C. elegans* [9], [35], the germline induces a signal through the somatic gonad that depends on the transcription factor *Ppa-daf-16* and the nuclear hormone receptor *Ppa-daf-12* when fed lab food *E. coli* or pathogenic bacteria. It must be noted however, that survival of the ablated *Ppa-daf-16* mutants is significantly greater than unablated *Ppa-daf-16* mutants (Supplementary Figure S1) (P<0.001), meaning that the ablation of these alleles still induces an increase in immune responsiveness. Hence, there must be another pathway acting in parallel. When the double mutant (*Ppa-daf-16*; *Ppa-daf-12*) is ablated there is no significant difference between *Ppa-daf-16* or *Ppa-daf-12*, demonstrating that in *P. pacificus* these genes are in the same pathway (data not shown), similar to *C. elegans* [57]. We conclude that somatic gonad signaling, causing increased longevity and pathogen resistance, is largely dependent on DAF-16/FOXO signaling in *P. pacificus*.

### Germline signaling genes regulate longevity and innate immunity

Aging affects susceptibility to diseases, vaccine failure, potentially autoimmunity and cancer as well as a decreasing the function of epithelial skin barriers, lung or gastrointestinal tract allowing pathogens to enter mucosal tissues, causing increased risk for aged innate immune systems [58]. We show that the increase in longevity and bacterial resistance upon germline ablation is an evolutionarily conserved response over the *Pristionchus* genus and, similar to the increased longevity and resistance of *C. elegans* germline deficient mutants to bacterial pathogens [9], [35], the response is strongly reliant on DAF-16/FOXO and DAF-12/NHR in *P. pacificus* [16]–[19]. Although the DAF-16 effect depends on the pathogen used in the analysis [16], DAF-16 regulates many stress-response genes including antimicrobial defenses [49], [59], [60] and is responsible for regulating the formation of stress resistant dauer juveniles [57]. We found an enrichment of genes regulated by DAF-16 associated with our enhanced pathogen phenotype e.g. *Ppa-lys-7*, *Ppa-clec-1*, *Ppa-clec-149*, *Ppa-clec-160* and *Ppa-clec-41*. Our study thus strongly indicates a conserved function of DAF-16/FOXO among nematodes, although it is clear that another pathway works in parallel with DAF-16 as our Z2 and Z3 ablated *Ppa-daf-16* (*tu302* and *tu901*) still showed an increase in resistance compared to un-ablated mutants. In *C. elegans* for example, it has been shown that in *glp* mutants the p38 MAPK pathway acts in parallel to DAF-16 [16]. Unfortunately however, we have no *P. pacificus* MAPK mutant so that it remains unclear whether MAPK signaling also acts in parallel to DAF-16/FOXO in *P. pacificus*.

The cell ablation data presented in this study indicate that the *P. pacificus* germline produces a signal that accelerates ageing [9] and depresses immunity to pathogens. We therefore argue that in wild type animals, the germline signals inhibit DAF-16 activity, but when Z2 and Z3 are ablated then the somatic gonad releases a signal and DAF-16 levels rise and regulate an abundance of genes that affect lifespan and bacterial resistance. The somatic gonad signal is poorly understood and how it is created, released or what the potential targets are remains elusive. It may indeed even be an emergency signal released upon removal of germline cells and secreted from injured cells to neighbors.

This is the first study to try to understand at the level of transcriptional response, how the reproductive system controls both immunity and longevity. Previous studies in *C. elegans* using RNAi screens have identified numerous genes and processes that affect the longevity of *glp-1* mutants including steroid hormone signaling [11], translation elongation [36], autophagy [12], oleic acid synthesis [13] and triglyceride metabolism [14]. By using a novel approach that combines experimental cell ablation, pathogen exposure and microarray analysis, we have expanded on this knowledge and shown an array of processes to be affected in germline-ablated animals. Many of these components have also been shown to be involved in longevity and immunity phenotypes in *C. elegans*, e.g proteasomal and cytoplasmic chaperonin function [16], [37], [38], [40]–[42], lipid metabolism [13], [14], [61], and translation initiation and elongation [36], [40], [43], [44]. In addition, we propose a role for disruption of nucleolar proteins (*nol* gene family) and nuclear pore complex proteins (*npp* gene family) in activating promoting longevity and pathogen resistance. This interpretation is also consistent with the recent reports suggesting that disruption of core cellular processes leads to activation of protective responses [40]. However, the mechanisms through which germline ablation affects these essential cellular processes remain to be discovered.

*C. elegans* mutants that exhibit remarkable lifespan extension e.g. *daf-2*, *age-1* and *glp-1* are also resistant to pathogens, oxidative and thermal stress [11], [16]–[19], [62]. Has *P. pacificus* evolved two separate pathways to enhance lifespan or increase immunity? The regulation of innate immunity and longevity by the *Cel-*DAF-2 insulin signaling pathway has caused many to think they are the same [63], [64] and studies have shown that longer lived *Caenorhabditis* species are more resistant to abiotic (heavy metals and heat shock) and biotic stresses (*P. aeruginosa* and *S. aureus*) [65]. However, there are a few examples in *C. elegans*, where contribution of genes towards either longevity or innate immunity could be separated. For example *sgk-1(ok538)* and *pdk-1(sa680)* mutants are long lived, but are not resistant to *P. aeruginosa* [17]. Similarly, loss of the GATA transcription factor ELT-2 enhances susceptibility to pathogens without shortening lifespan [66] and *sek-1(km4)* and *pmk-1(km25)* mutants are hypersusceptible to pathogens, but have a relatively normal lifespan [67], [68]. In our experiments comparing germline-ablated animals fed either *S. marcescens* or *E. coli* OP50, we see only 244 differentially expressed genes comprising of lectins and lipid metabolism genes. Similarly, the expression profile E4 of ablated versus unablated animals on *S. marcescens* was very similar to profile E1 (ablated versus unablated animals on *E. coli*), with only 292 genes specific to E4. With such a small number of genes it seems likely that longevity and pathogen resistance are regulated in a similar manner, although the small differences might be functionally important and could be contributed by a parallel pathway.

Taken together, manipulations of the gonad in an array of diverse organisms such as nematodes [9], flies [10], mice [6], [7] and humans [8] have demonstrated increases in lifespan but the mechanisms have remained elusive. By taking a combined approach of using laser microsurgery, pathogen exposure and whole genome microarrays we have demonstrated that upon germline ablation *P. pacificus* can live longer and resist pathogens by regulating numerous downstream effectors that affect an array of processes including translation initiation factors in the ribosome, proteasome maintenance, insulin signaling, nuclear pore complexes and lipid metabolism, which is dependent on the transcription factor DAF-16. It has been well documented that insulin signaling and DNA modifications in FOXO affect longevity in humans [69], but little is known about the role and contribution of genes involved in immunity. We show that processes integral for increasing lifespan and enhancing innate immunity are largely similar. Therefore, upregulation of pathogen defense systems might be an essential factor for living longer.

## Materials and Methods

### Nematode and bacteria strains

*P. pacificus* WT RS312, RS5160, S264, M2, *Pristionchus* sp. 3, *Pristionchus* sp. 16, *Ppa-daf-16* (*tu302* and *tu901*) and *Ppa-daf-12* (*tu390* and *tu389*) were maintained on 5 cm NGM agar plates laced with *E. coli* (strain OP50) at 20°C. *S. marcescens* strain C2 was isolated from an *Oryctes* beetle from La Reunion and *X. nematophila* strain XN2 was a gift from Becker Underwood, U.K. and were maintained on LB plates.

### Survival assays and analysis

Bacteria (*S. marcescens* and *X. nematophila* XN2) were grown in LB at 30°C overnight in a shaking incubator. The following day 100 µl were spread evenly onto predried 5 cm NGM plates and incubated overnight at 30°C. Three independent biological replicates of 20 worms per plate were exposed to either pathogen or *E. coli* OP50 and were monitored for survival. Worms which failed to respond to a touch of the worm-pick were considered dead. Survival of *P. pacificus* fed *E. coli* OP50 or pathogens was compared using the log rank test.

### Cell ablation and RNA collection

*P. pacificus* J2 stage were picked into 2.8 µl PBS on a agar pad containing 1 mM NaN_3_. Ablations would take place within 1 hour of hatching at 20°C. After ablation nematodes were stored at 20°C and successful ablation was verified 48 hours later. Nematodes unablated were grown in parallel and acted as controls. For microarrays 20 *P. pacificus* (either Z2 or Z3 ablated or unablated) were picked onto 5 separate NGM plates either spread with *E. coli* OP50 or *S. marcescens* and incubated at 25°C for 4 hours. Nematodes were then picked into 500 µl of Trizol and stored at −80°C until further analysis. The treatments therefore included (i) Z2 and Z3 ablated *P. pacificus* fed *E. coli* OP50 (ii) Z2 and Z3 ablated *P. pacificus* fed *S. marcescens* (iii) unablated *P. pacificus* fed *E. coli* OP50 (iv) unablated *P. pacificus* fed *S. marcescens*. Development of ablated and unablated *P. pacificus* fed both *E. coli* OP50 and *S. marcescens* was the same. Each treatment consists of a pool of approximately 100 animals and there were 4 biological replicates of each such pool.

### Microarray experiments

A total of 8 microarray hybridizations were carried out for two of the comparisons (E1, E2) depicted in Figure 4. The third comparison (E3, transcriptional response of wild type *P. pacificus* to *S. marcescens*) has been previously characterized in our lab (AS, RR, II and RJS, unpublished data). The fourth comparison E4 (Ablated versus unablated animals on *S. marcescens*) could be computationally derived from this experimental design as the contrast E2+E1−E3. We used our custom designed oligonucleotide microarrays manufactured by Agilent Technologies, which contain ∼93,000 unique probes for the ∼23,000 *P. pacificus* predicted genes (NCBI GEO accession GPL14372, see [26] for design details of custom microarrays). Equal amounts of total RNA (500 ng to 800 ng) from four biological replicates of each experimental and control samples was used to produce Cy5 or Cy3 dye labeled cRNA using Quick Amp Labelling Kit (Agilent Technologies Inc., USA) as per manufacturer's instructions. Depending upon the amount of total RNA used, appropriate amounts of positive control RNA (Spike Mix-A and Spike Mix-B, from Agilent Technologies) were added to the mix before reverse transcribing the total RNA, as per manufacturer's instructions. The experiments were carried out in a two-color format where Cy5 and Cy3 dye labeled cRNA from experimental and control sample is co-hybridized on the same microarray. The four biological replicates per experiment included two dye-swap experiments to account for differences in dye labeling. Hybridization and washing of the arrays was carried according to manufacturer-supplied protocol. The arrays were scanned on a GenePix 4000B Microarray Scanner, and raw data extracted using GenePix Pro software (version 6).

### Microarray data analysis

We used the Bioconductor package limma [70] for analysis of our microarray data. Array quality was checked for parameters such as uniform background and foreground intensities over the entire array. The raw signal was background corrected using the normexp method [71] and the arrays were then lowess normalized individually (“normalizeWithinArrays” option), with differential weights assigned to probes and to positive control spike-ins, which are expected to show no fold change [72]. This differential weighing of probes is particularly necessary to account for differences in relative proportion of mRNA versus total RNA, and/or differences in the amount of RNA produced per worm under different experimental conditions. Without this differential weighing scheme, the fold change calculations can be erroneous [26], [73]. The weight parameters were optimized based on MA-plots such that spike-in controls show their expected fold change values. lmFit function was used to fit a linear model to probe intensities across arrays, differential expression was calculated by empirical Bayes method using the eBayes function [74], and control of FDR was employed as the multiple testing correction. MA-plots were also used as diagnostic to identify and remove outlier arrays before fold-change calculations, such that at least three biological replicates were used for each experiment. Further data analysis was carried out using custom scripts in Perl and the statistical package R. Raw and processed data from all the experiments from this publication have been deposited in a MIAME compliant format at NCBI's Gene Expression Omnibus database (http://www.ncbi.nlm.nih.gov/geo/), with accession numbers GSE37331 and GSE3733. Expression data from our as yet unpublished experiments (AS, RR, II and RJS, unpublished data, in submission) comparing transcriptomes of wild type *P. pacificus* exposed to *S. marcescens* versus *E. coli* OP50 are available under accession number GSE36521.

### Functional analysis of microarray data

We have previously used a pairwise best BLASTP strategy to identify 7,176 pairs of orthologs in *C. elegans* and *P. pacificus* [26]. Probes for 6,126 of these gene pairs exist on microarrays of *P. pacificus*. Gene Ontology annotations were transferred to *P. pacificus* genes using these orthology relations and topGO tool was used for enrichment analysis [75]. Pfam domain annotations are the same as described before [26]. For enrichment analysis, only the domains for which minimum 5 protein coding genes were represented on each microarray were used. Statistical significance of enrichment of Pfam domains in each expression profile determined using a 2×2 Fishers exact test, at a p-value cut-off of 0.05. Expression cluster data from relevant experiments [26], [49]–[53] was compiled from WormBase or from Supplementary Materials of the respective publications. When needed, we inferred expression clusters for *P. pacificus* from *C. elegans* datsests based on the set of orthologs. For the enrichment analysis, only genes with at least one expression-cluster annotation were used as the background set. P-values for expression cluster enrichment in each expression profile were computed with a 2×2 Fisher exact test with a p-value cut-off of 0.05 as the significance threshold.

## Supporting Information

Figure S1 **Effect of Z2 and Z3 germline ablation on survival of** ***P. pacificus*** **insulin signaling mutants exposed to** ***S. marcescens*** **.** Survival of *Ppa-daf-16* (*tu901*) Z2 and Z3 ablated (blue) and un-ablated (red), and *Ppa-daf-16* (*tu302*) Z2 and Z3 ablated (yellow) and un-ablated (green) exposed to *S. marcescens.* Error bars represent ± S.E.M.
(TIFF)

Figure S2 **Comparison of fold-changes in expression profiles E4 (ablated versus unablated animals exposed to** ***S. marcescens*** **) and E1 (ablated versus unablated animals exposed to** ***E. coli*** **).** The two profiles are quite similar, with almost similar fold-changes for the majority of the genes. (Pearson correlation = 0.90, Spearman's rank correlation = 0.89).
(TIFF)

Table S1 **Summary statistics of** ***P. pacificus*** **ablation experiments monitoring survival when fed** ***S. marcescens*** **and** ***X. nematophila*** **.** Mean survival and standard errors for all conditions tested, and p-values from log Rank test assessing significance of difference between various comparisons. The rows 8 and 9 (marked with an “*”) correspond to the pathogen *X. nematophila*.
(TIFF)

Table S2 **Genes significantly differentially expressed in the comparison of germline-ablated animals fed on** ***E. coli*** **OP50 versus un-ablated animals fed** ***E. coli*** **OP50 (Expression profile E1).** Processed microarray data for experiment E1 for each *P. pacificus* gene with its log2 fold change, FDR corrected p-value and average expression value (log2 scale).
(XLS)

Table S3 **Genes significantly differentially expressed in the comparison of germline-ablated animals fed on** ***S. marcescens*** **versus germline-ablated animals fed** ***E. coli*** **OP50 (Expression profile E2).** Processed microarray data for experiment E2 for each *P. pacificus* gene with its log2 fold change, FDR corrected p-value and average expression value (log2 scale).
(XLS)

Table S4 **Enrichment for GO terms from categories (a) Biological Process (b) Molecular Function (c) Cellular Component, in genes differentially regulated upon germline ablation.** The total number of genes in *P. pacificus* genome with a given GO term are in the “Annotated” column, the number of genes observed to be significantly differentially expressed are in the column “Significant” and the number of genes expected by random chance are given in the column “Expected”. The p-value for enrichment was calculated using the method “elimFisher” in the “topGO” tool in Bioconductor.
(XLS)

Table S5 **Pfam domains enriched in the genes regulated upon germline ablation.** The total number of genes in *P. pacificus* genome whose products contain a given Pfam domain are in the “Total” column, the number of genes observed to be significantly differentially expressed are in the column “Observed” and the number of genes expected by random chance are given in the column “Expected”. “Enrichment” is the ratio of Observed to Expected. P-values for enrichment are from a 2×2 Fisher's Exact test and corrected for False Discover Rate. Proteasome/Ubiquitin system related domains are highlighted in orange. Domains involved in RNA metabolism are highlighted in blue.
(XLS)

Table S6 **Genes common between pathogen response of germline-ablated animals (experiment E2) and pathogen response of un-ablated animals (experiment E3).** About 100 significantly differentially expressed genes are common between the expression profiles E2 and E3. All genes except one show a similar direction of fold change. The corresponding ortholog in *C. elegans* exists for only 30 of these genes.
(XLS)

Table S7 **Genes significantly differentially expressed in the comparison of germline-ablated animals fed on** ***S. marcescens*** **versus un-ablated animals fed** ***S. marcescens*** **(Expression profile E4).** Processed microarray data for experiment E4 for each *P. pacificus* gene with its log2 fold change, FDR corrected p-value and average expression value (log2 scale).
(XLS)

Table S8 **List of the 292 genes exclusive to expression profile E4 (ablated versus unablated animals exposed to** ***S. marcescens*** **) in a comparison against profile E1 (ablated versus unablated animals exposed to** ***E. coli*** **).** The log2 fold change, FDR corrected p-value and average expression value (log2 scale) of the 292 genes exclusive to profile E4.
(XLS)

Table S9 **Microarray expression clusters showing significant overlap with genes up or down regulated in expression profile E4 (ablated versus unablated** ***P. pacificus*** **exposed to** ***S. marcescens*** **).** The profile E4 is also enriched for genes that are known targets of DAF-16, and TGF-beta pathway in *C. elegans*, and genes regulated in response to various pathogens in *P. pacificus*. Significance scores are −log10 of the p-values obtained in a 2×2 Fisher's exact test, with a zero value indicating non-significant enrichment. The results are qualitatively very similar to that seen for profile E1 (compare with Table 1).
(XLS)
