# Supplementary figures and images for: Genome-Wide Analysis of Germline Signaling Genes Regulating Longevity and Innate Immunity in the Nematode Pristionchus pacificus
Source: PLoS Pathog. 2012 Aug 9;8(8):e1002864. doi: 10.1371/journal.ppat.1002864 (PMC3415453; doi:10.1371/journal.ppat.1002864)

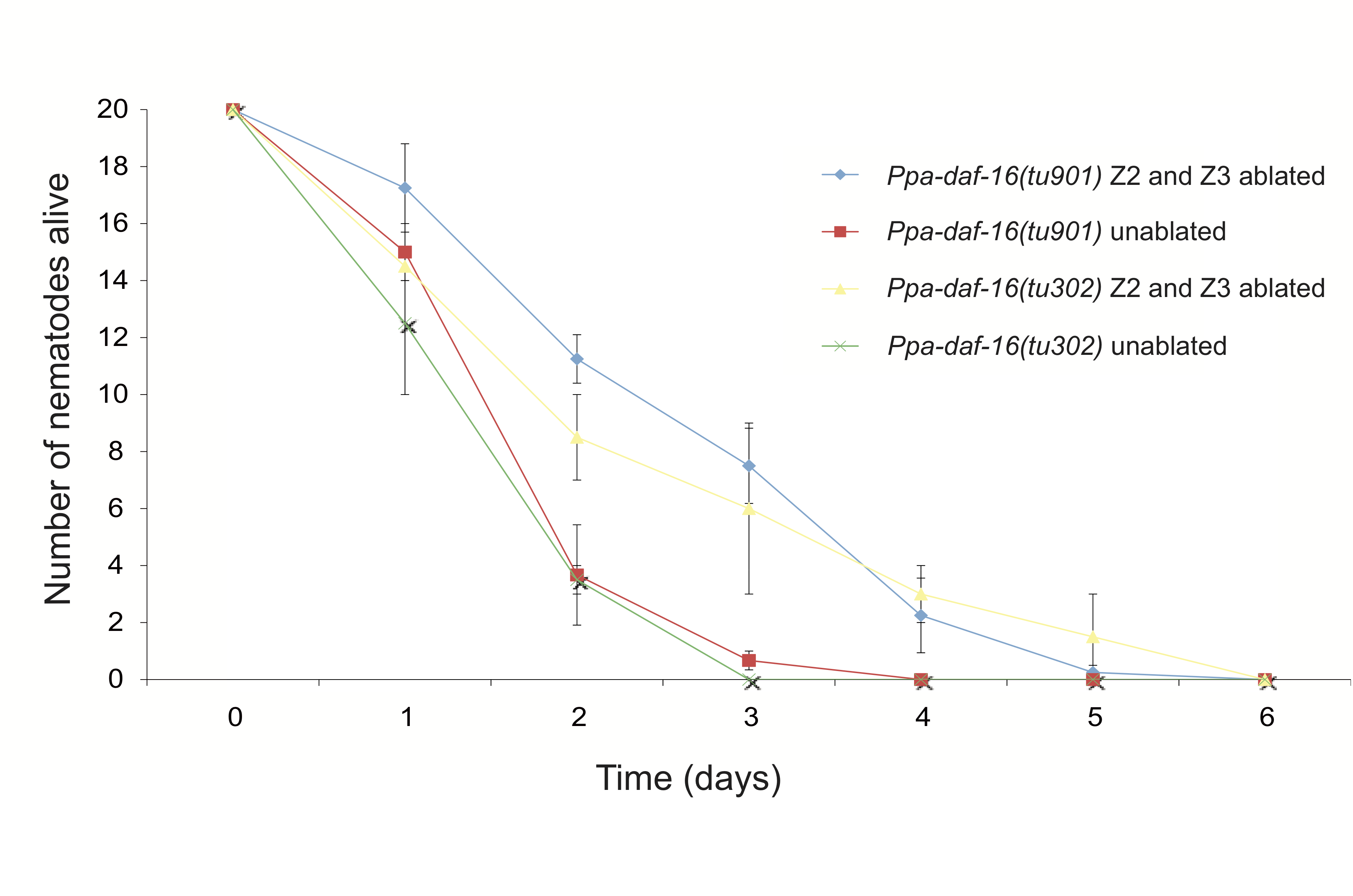

Supplement: Figure S1 — Effect of Z2 and Z3 germline ablation on survival of P. pacificus insulin signaling mutants exposed to S. marcescens . Survival of Ppa-daf-16 (tu901) Z2 and Z3 ablated (blue) and un-ablated (red), and Ppa-daf-16 (tu302) Z2 and Z3 ablated (yellow) and un-ablated (green) exposed to S. marcescens. Error bars represent ± S.E.M. (TIFF) [file ppat.1002864.s001.tiff]

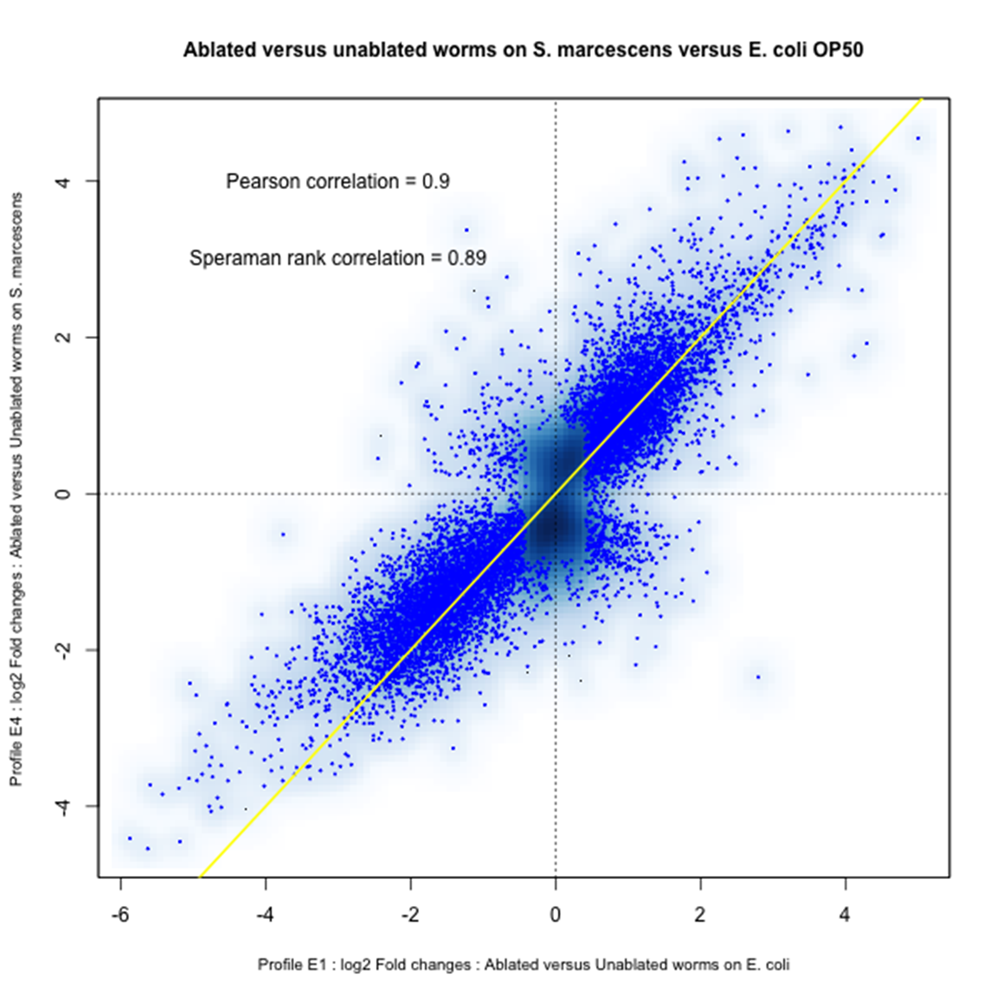

Supplement: Figure S2 — Comparison of fold-changes in expression profiles E4 (ablated versus unablated animals exposed to S. marcescens ) and E1 (ablated versus unablated animals exposed to E. coli ). The two profiles are quite similar, with almost similar fold-changes for the majority of the genes. (Pearson correlation = 0.90, Spearman's rank correlation = 0.89). (TIFF) [file ppat.1002864.s002.tiff]

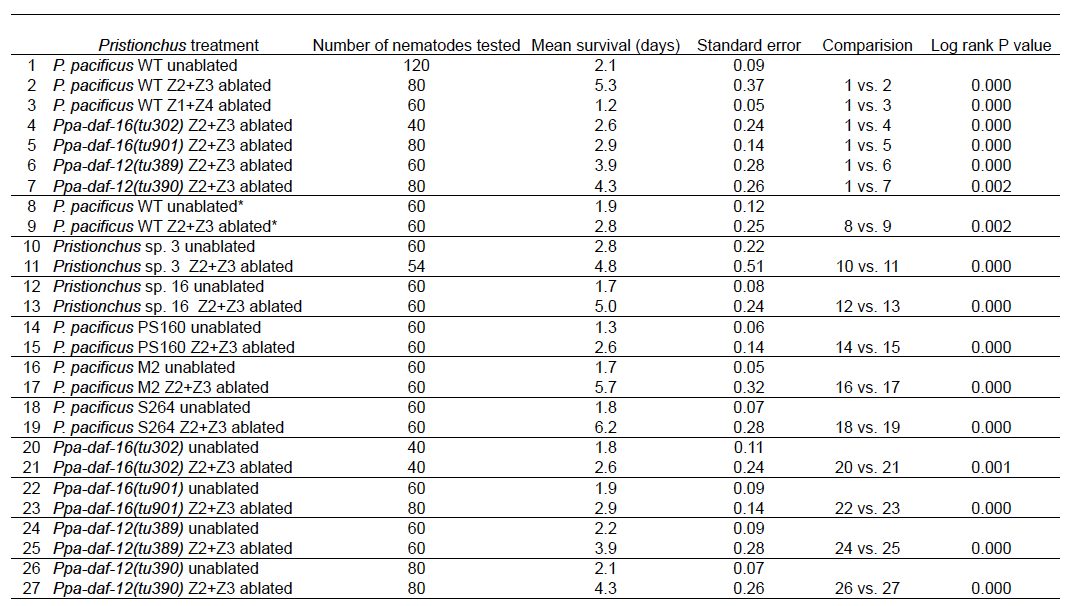

Supplement: Table S1 — Summary statistics of P. pacificus ablation experiments monitoring survival when fed S. marcescens and X. nematophila . Mean survival and standard errors for all conditions tested, and p-values from log Rank test assessing significance of difference between various comparisons. The rows 8 and 9 (marked with an “*”) correspond to the pathogen X. nematophila. (TIFF) [file ppat.1002864.s003.tiff]
